# Editorial

**Published:** 1840-02-29

**Authors:** 


THE MEDICAL EXAMINER.

PHILADELPHIA, FEBRUARY 29, 1840.

### CONTINUED FEVERS OF GREAT BRITAIN.

We commence the publication of a series of cases relative to the continued fevers of Great

Britain. These were taken at London, by Dr. Shattuck, in a late visit to England. Feeling, as we do, no little interest in the matter, we are glad to bring the subject to the notice of the profession. There is still so much confusion in the nomenclature of two diseases which are essentially different, that the mind of the student is distracted, and he is at loss to reconcile the description of the disease given by the British writers with that of the French pathologists. The same appellation is commonly given to the two diseases, and hence the error naturally enough arises.

The present state of our knowledge upon this subject, may be explained in a few words. The disease which is described by the French writers, Petit and Serres, Louis and Chomel, is the more constant disorder ; it is generally sporadic, but it occasionally assumes an epidemic character. It does not appear to be limited to any particular country, nor to any season. It is most frequent in New England, but extends over the whole United States ; as a general rule, it prevails most, where malarious diseases are least frequent. This disease is not only characterized by fixed symptoms, but by fixed anatomical lesions, which consist in an inflammation of the follicles of the small intestine, and of the mesenteric glands connected with them, and in softening of the spleen. This form of fever we call typhoid, and apply that term to none other.

The typhus fever is a disease much more frequent in the British islands than in any other country ; it is at times very severe, and attended with symptoms of extreme prostration of the nervous power, and decided alterations of the blood. This is especially the case when the disease attacks men crowded together in confined situations, such as jails and camps. It then is highly contagious, and is called jail, camp, or ship fever. If the typhoid fever happens to prevail in the same localities, it is often improperly confounded under similar vague denominations. At other times, the disease is much milder, the mortality very slight ; it is then properly termed typhus mitior. In neither of these varieties of typhus is there any fixed local lesion ; the disease does not differ in this respect from the proper exanthemata, with which it offers a number of points of resemblance.

The cause of the confusion of ideas in relation to the two diseases, is just this : Physicians have attempted to unite the symptoms of two different disorders. Hence the descriptions found in the works of one writer, were inapplicable to those of another. The true appreciation of the symptoms is readily made by recollecting, that although both forms of fever are often found together in the same locality, they may always be separated, and the symptoms and lesions will then be found widely different.

